# Supplementary material for: Circulating extracellular vesicles in plasma carry accessible molecular signatures of aging in mice
Source: bioRxiv. 2026 Jul 10:2026.07.10.737625. Preprint. [Version 1] doi: 10.64898/2026.07.10.737625 (PMC13370508; doi:10.64898/2026.07.10.737625)
Supplement: Supplementary file 1 [file media-2.pdf]

Suplementary Material for Manuscript

Title

Circulating extracellular vesicles in plasma carry accessible molecular signatures of aging in mice

Authors

Kristine A. Tsantilas[1], Michael Riffle[1], Gennifer E. Merrihew[1], Christine C. Wu[1], Gregory R. Keele[2], Aaron Maurais[1], Richard S. Johnson[1], Alison Luciano[2], Laura Robinson[2], Gary A. Churchill[2], Michael J. MacCoss[1,†]

Affiliations

1. Department of Genome Sciences, University of Washington, 1705 NE Pacific Street, Seattle, Washington 98195, United States
2. The Jackson Laboratory, 600 Main Street, Bar Harbor, Maine, 04609, United States

† Corresponding author

Contents

Supplementary Figures 2

Supplementary Figures

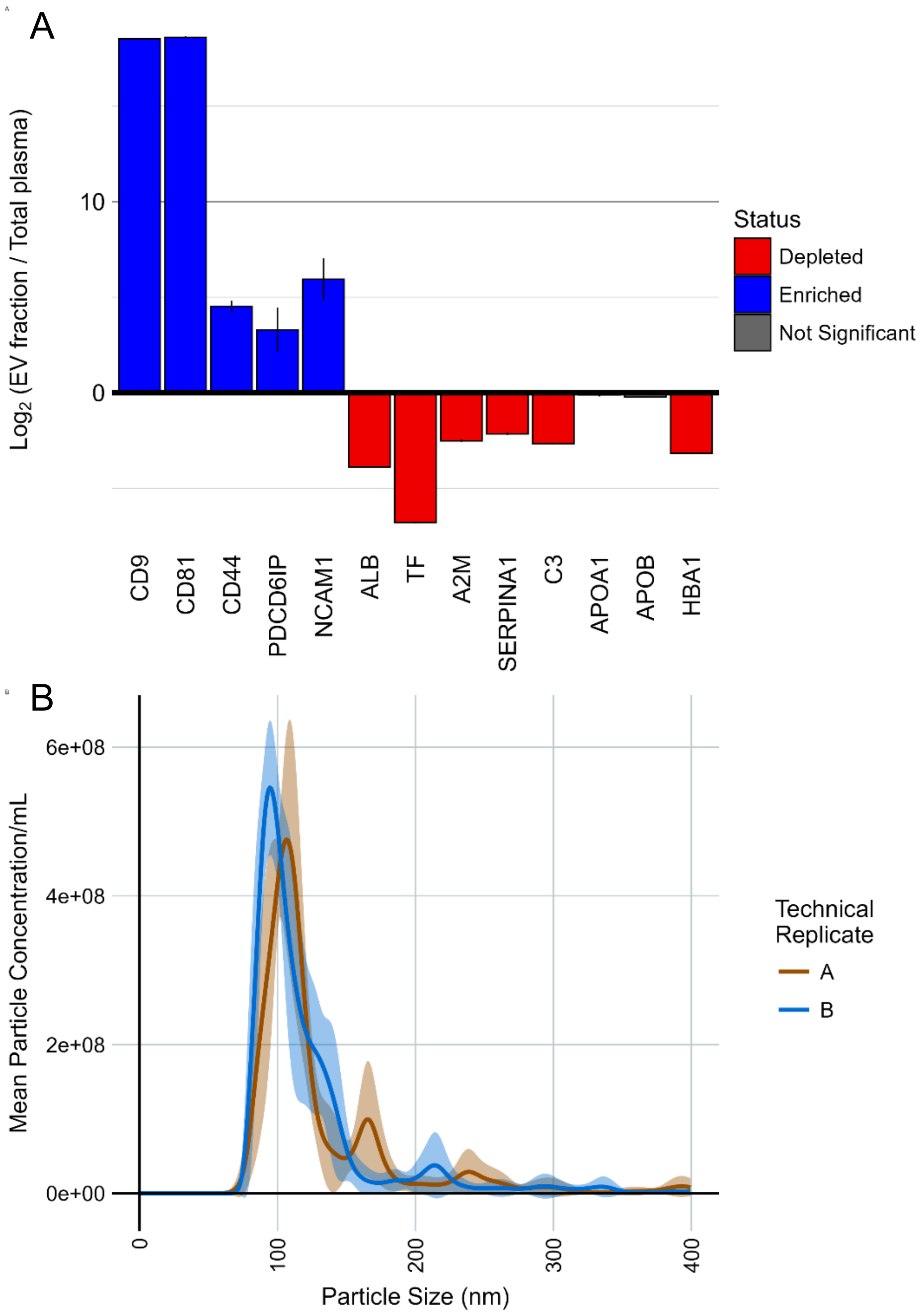

Supplementary Figure 1: We confirmed that like previous studies in human plasma the Mag-Net protocol shows an enrichment of established EV markers (A) and a depletion of many high abundance proteins (B) that comprise the majority of the plasma proteome's mass. Nanosight particle analysis also confirmed the presence of particles isolated using Mag-Net (C).

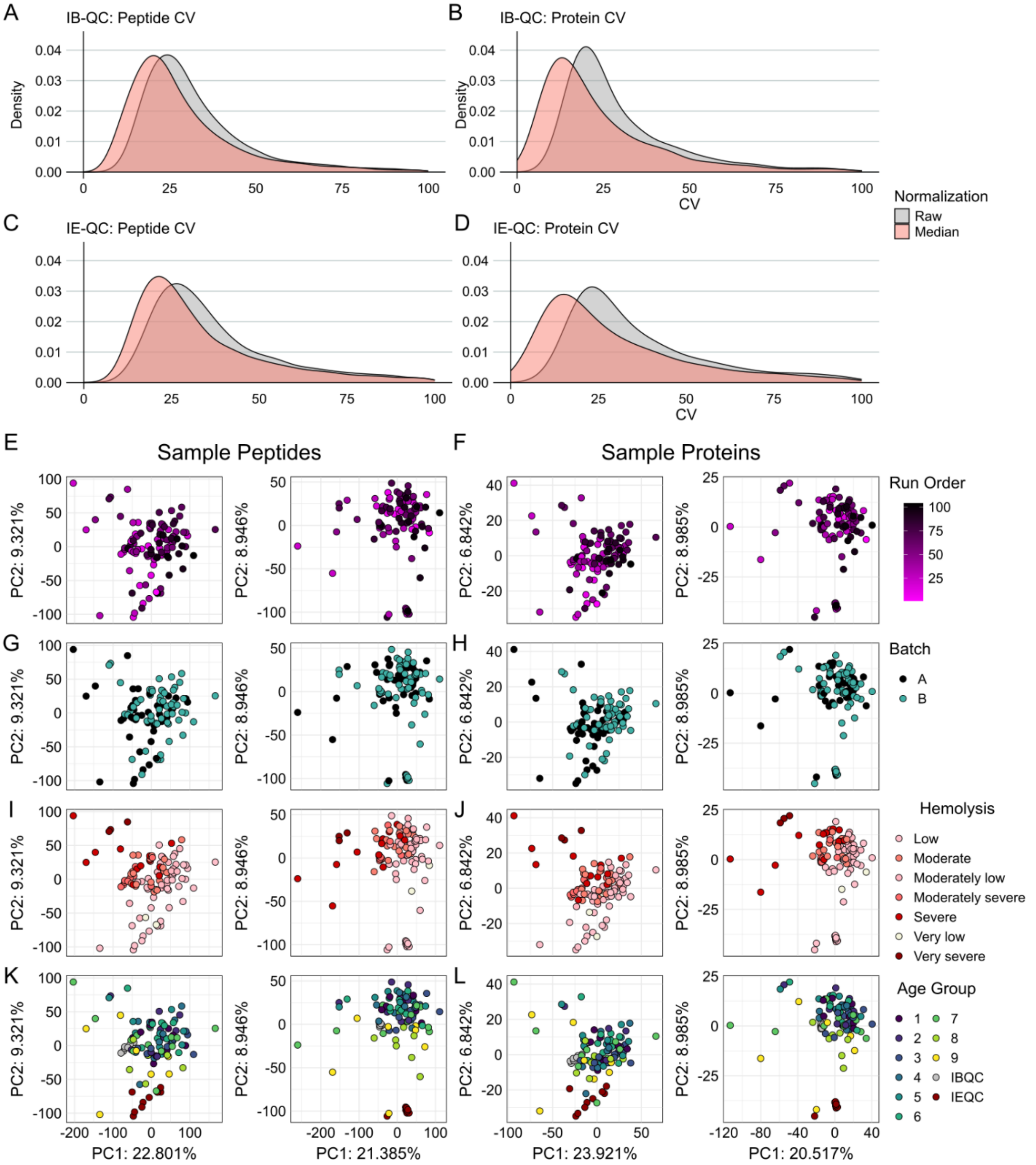

Supplementary Figure 2: The distribution of CVs of the peptides and proteins detected in the raw and median-normalized data were evaluated in the IB-QC and IE-QC. Median normalization shifted the distribution of CVs lower in the peptides (A) and proteins (B) of the IB-QC sample and in the peptides (C) and proteins (D) of the IE-QC sample. PCA plots colored by different variables highlighted a few points. First, we found no evidence of a run order effect at the peptide (E) or protein-level (F) with or without median normalization. There was not clear evidence of a sample preparation batch effect with or without median normalization at the

peptide (G) or protein (H) level. Some hemolyzed samples were present, and could be observed in at the peptide (I) and protein (J) level. The IB-QC was more hemolyzed than the IE-QC, and this was visualized in a cluster in (I) and (J). Finally, when colored by QC type and by the age groups (bins) described in Figure 1, we observed clear clustering of each control together in the raw data that became tighter after median normalization at the peptide (K) and protein (L) level. We also saw increasing spread of the data with increasing age. The youngest mice in the lowest age group numbers tended to be more tightly clustered than the oldest mice in the highest age group numbers. This increased spread with age was better visualized after median normalization in peptides (K) and proteins (L).

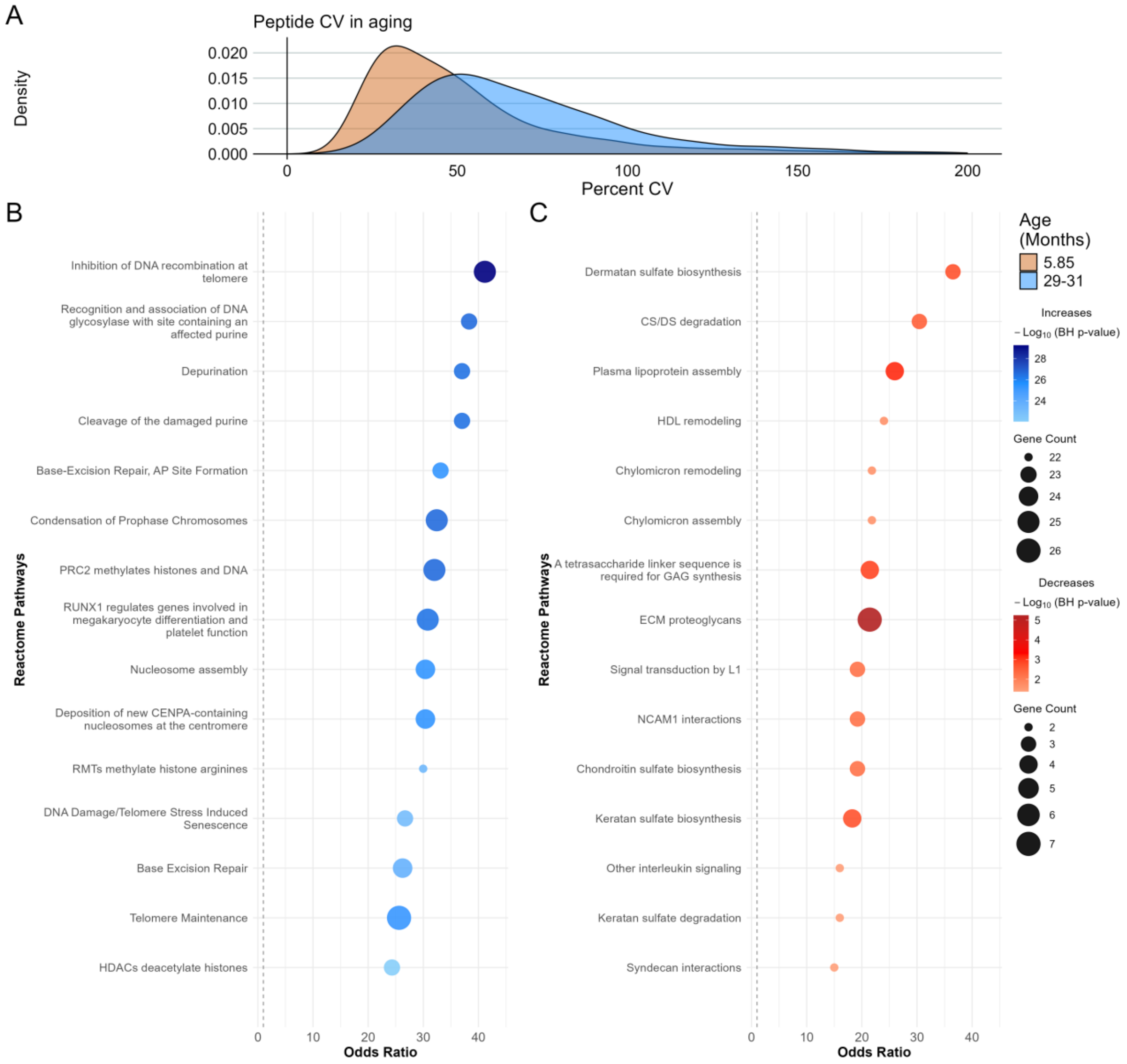

Supplementary Figure 3: Density plot of the peptide CV for mice in the 5.5 month (orange) and 31 month (blue) age group indicates that there is more variability in aged mouse peptide abundance (A). Spearman correlation was applied to extract proteins where their abundance increased or decreased with increasing age, and Reactome pathways associated with proteins that correlated positively (B) and negatively (C) were evaluated using the ReactomePA R package. The top 15 pathways with BH-corrected p-value < 0.05 and the highest Odds Ratios are plotted.

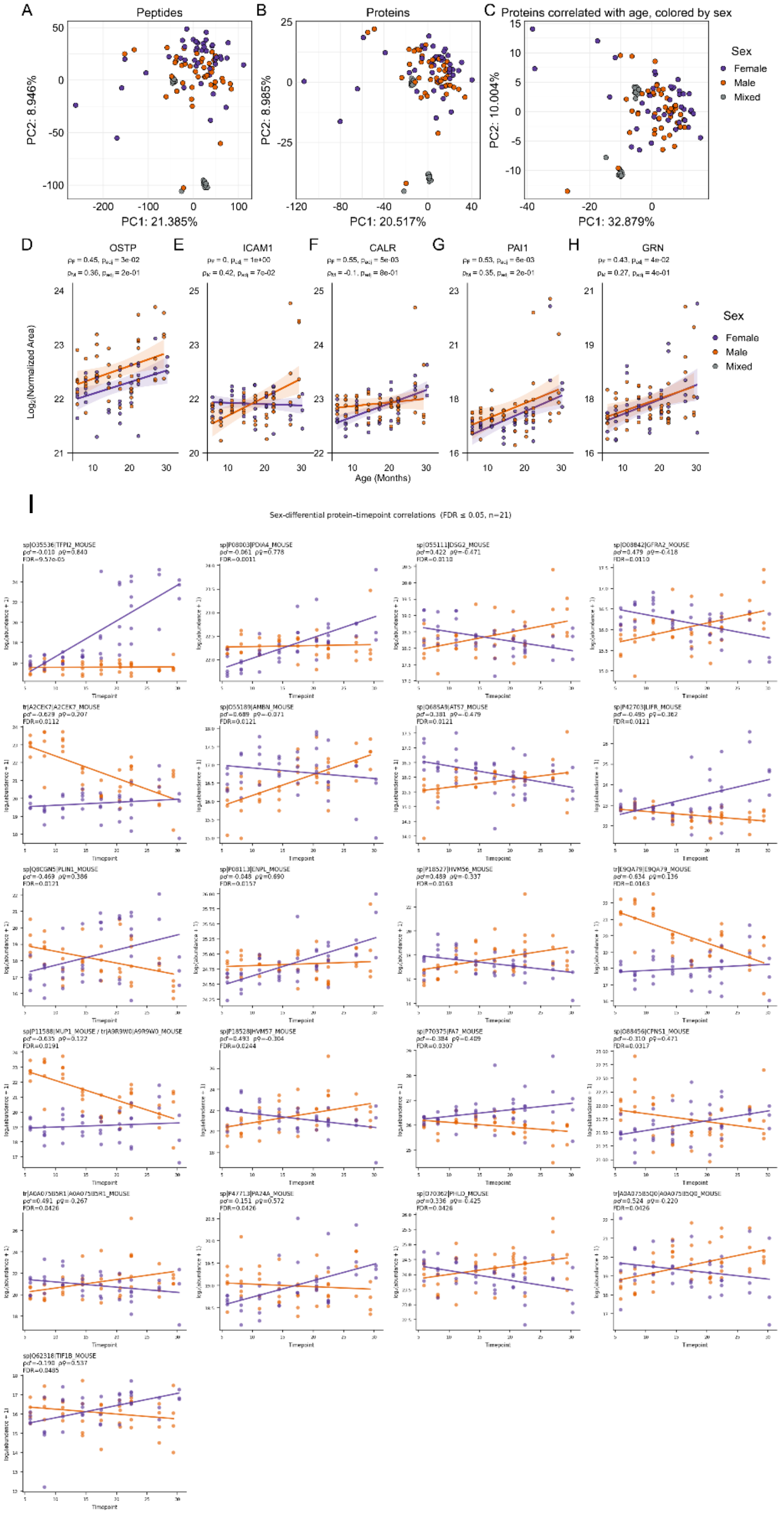

Supplementary Figure 4: PCA at the peptide level (A). PCA at the protein level (B). PCA at the protein level filtering the dataset for the proteins with the Spearman Rho greater than 0.3 or less than -0.3 with a p-adjusted < 0.05 after Benjamini-Hochberg correction (C). After re-calculating Spearman correlation at the protein-level for each sex separately, the same known age and senescence-related markers highlighted in Figure 2E are plotted in (D), (E), (F), (G), and (H) split by sex. There are 21 proteins with p < 0.05 after multiple hypothesis testing correction that have the largest difference in Spearman Rho with increasing age between males and females (I). Males and females are plotted in orange and purple, respectively. We used OLS to examine what proteins had a sex-specific age and found 16 had a significant q-value after FDR correction. This included 10 proteins that overlapped with the Spearman analyses.

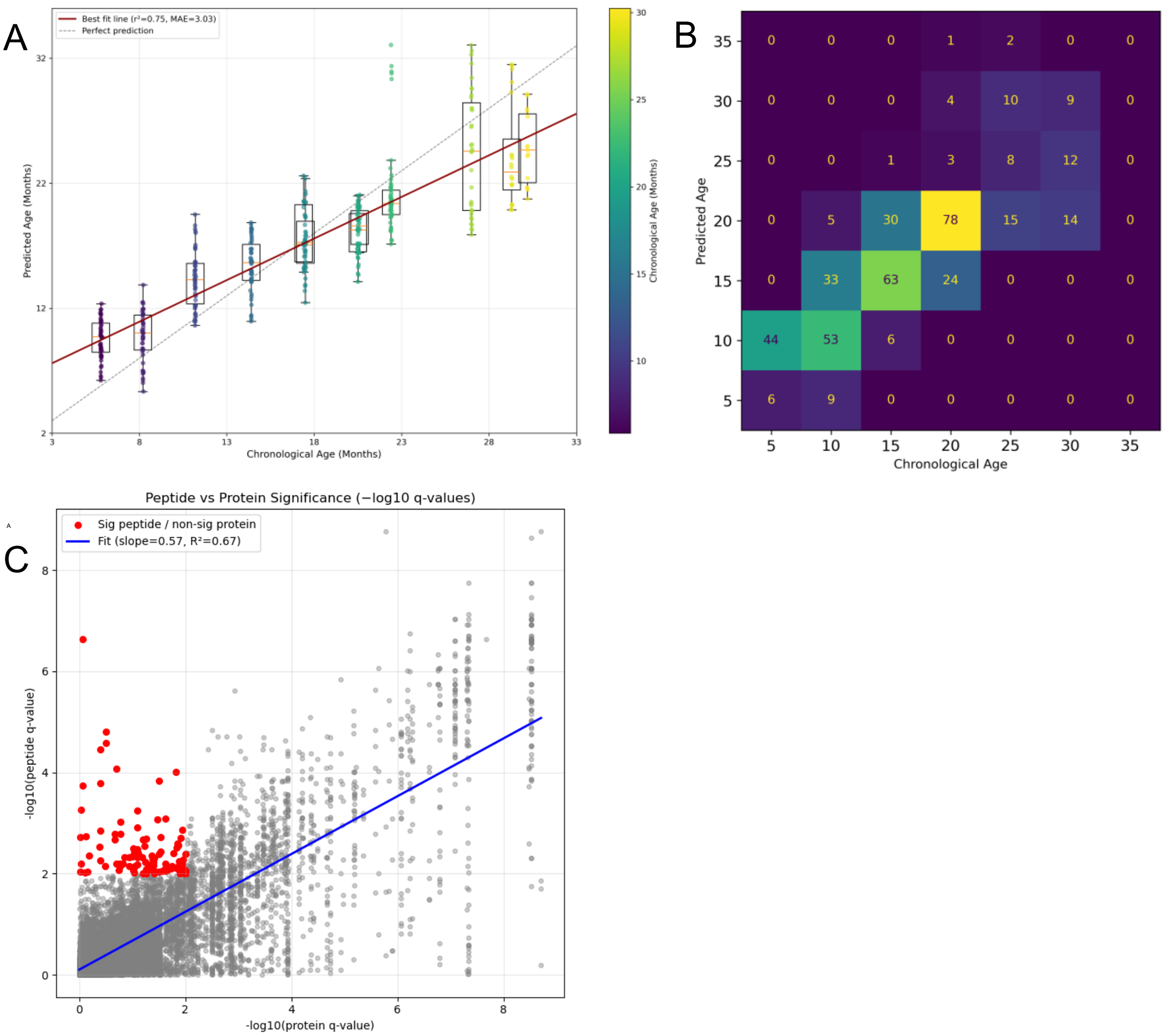

Supplementary Figure 5: Plot of the 10-fold cross-validation with 5 repeats of the Elastic Net model to predict chronological age using the median-normalized, logarithmically-transformed peptide abundances measured in the full dataset of 15,969 peptides (A). The confusion matrix of this model (B) illustrates that the model performs best in the middle of the age-range tested and is less accurate at predicting chronological age at the earliest and latest ages of the cohort. The q-values of peptides from the peptide-level model are plotted on the y-axis against the q-values proteins from the protein-level model (C). There were 96 peptides that reached significance ($q < 0.01$) in the peptide-level model with proteins that were not significant in the protein-level model.
